# N6-methyladenosine modification of *PLOD2* causes spermatocyte damage in rats with varicocele

**DOI:** 10.1186/s11658-023-00475-4

**Published:** 2023-09-05

**Authors:** Huan Li, Jun Zhao, Hao Deng, YuCheng Zhong, Mian Chen, LinSheng Chi, GuoQun Luo, Cong Cao, Cong Yu, Honghai Liu, Xinzong Zhang

**Affiliations:** 1 Assisted Reproductive Technology Center, Foshan Maternal and Child Health Care Hospital, Foshan, China; 2 Pharmacy Department, Foshan Maternal and Child Health Care Hospital, Foshan, China; 3 Assisted Reproductive Technology Centre, Maternity and Child Healthcare Hospital of Meizhou, Meizhou, China; 4 NHC Key Laboratory of Male Reproduction and Genetics, Guangdong Provincial Reproductive Science Institute (Guangdong Provincial Fertility Hospital), Guangzhou, China

**Keywords:** PLOD2, m^6^A, Varicocele, GC-2 cell, Rat

## Abstract

**Background:**

In recent years, N6-methyladenosine (m^6^A) methylation modification of mRNA has been studied extensively. It has been reported that m^6^A determines mRNA fate and participates in many cellular functions and reactions, including oxidative stress. The *PLOD2* gene encodes a protein that plays a key role in tissue remodeling and fibrotic processes.

**Methods:**

The m^6^A methylation and expression levels of *PLOD2* were determined by m^6^A methylated RNA immunoprecipitation sequencing (MeRIP-seq) and MeRIP-quantitative polymerase chain reaction (qPCR) in the testes of varicocele rats compared with control. To determine whether *IGF2BP2* had a targeted effect on the *PLOD2* mRNA, RNA immunoprecipitation-qPCR (RIP-qPCR) and luciferase assays were performed. CRISPR/dCas13b-ALKBH5 could downregulate m^6^A methylation level of *PLOD2*, which plays an important role in *PLOD2*-mediated cell proliferation and apoptosis in GC-2 cells.

**Results:**

*PLOD2* was frequently exhibited with high m^6^A methylation and expression level in the testes of varicocele rats compared with control. In addition, we found that *IGF2BP2* binds to the m^6^A-modified 3′ untranslated region (3′-UTR) of *PLOD2* mRNA, thereby positively regulating its mRNA stability. Targeted specific demethylation of *PLOD2* m^6^A by CRISPR/dCas13b-ALKBH5 system can significantly decrease the m^6^A and expression level of *PLOD2*. Furthermore, demethylation of *PLOD2* mRNA dramatically promote GC-2 cell proliferation and inhibit cell apoptosis under oxidative stress.

**Conclusion:**

As a result, we found that varicocele-induced oxidative stress promoted *PLOD2* expression level via m^6^A methylation modification. In addition, targeting m^6^A demethylation of *PLOD2* by CRISPR/dCas13b-ALKBH5 system can regulate GC-2 cell proliferation and apoptosis under oxidative stress. Taken together, our study has acquired a better understanding of the mechanisms underlying male infertility associated with oxidative stress, as well as a novel therapeutic target for male infertility.

**Graphical Abstract:**

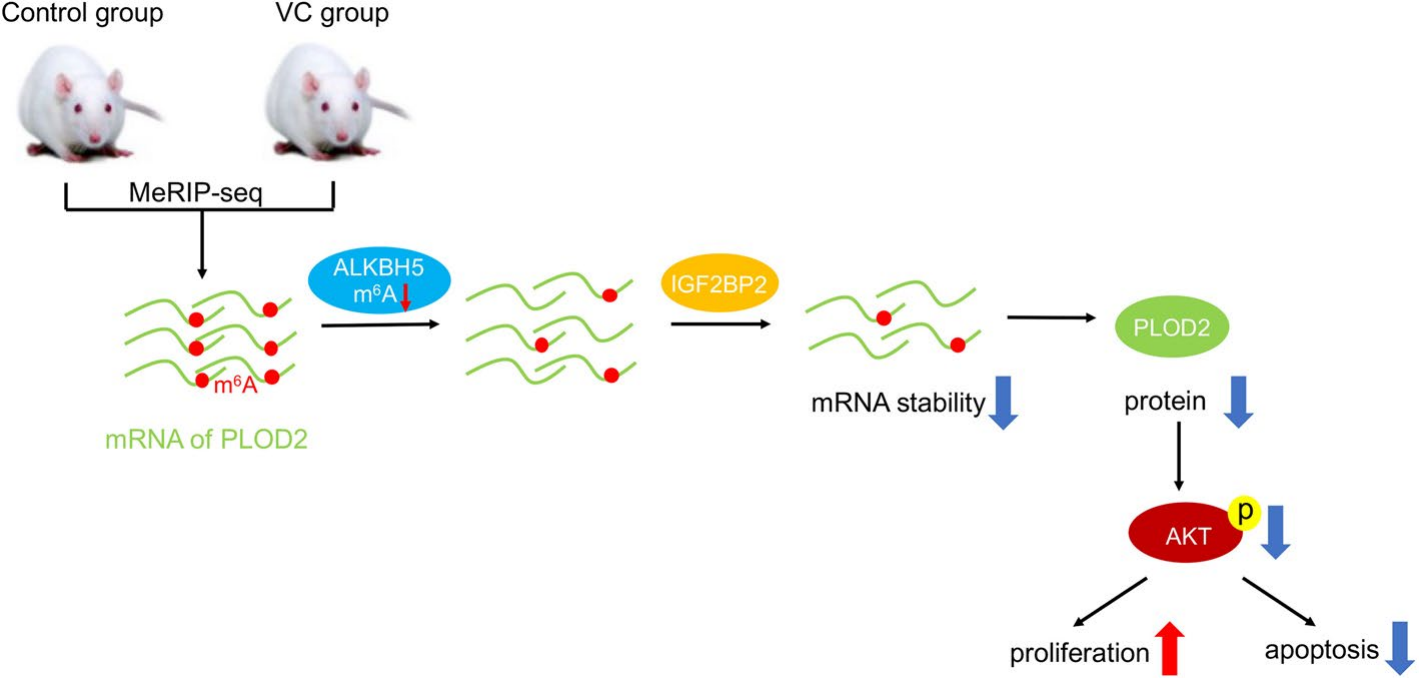

**Supplementary Information:**

The online version contains supplementary material available at 10.1186/s11658-023-00475-4.

## Background

The incidence of male infertility has gradually increased in recent years, which is affecting families around the world. Worldwide, male reproductive health is recognized as an important issue. Varicocele (VC) is a pathological occurrence characterized by elongation, expansion, and tortuousness of male spermatic veins. The incidence rate of varicocele in young male urology is about 15%, and it is mainly determined by its physiological and anatomical factors [1]. With a clear diagnosis of VC, infertility can be as high as 35%, which is a major cause of male infertility [2]. The relationship between VC and male infertility has been studied extensively in recent years, resulting in a wide range of theories [3]. VC may induce male infertility through the effects of testicular microcirculation, vasoactive substances, oxidative stress, nitric oxide, hypoxia, immunity, and apoptosis.

VC is caused by blocked spermatic vein returns, which decrease blood flow to the testes and makes the testes hypoxic, ultimately resulting in a decrease in sperm production. Based on the studies of Kilinc et al., VC is associated with tissue hypoxia and related pathophysiological processes, thereby affecting spermatogenesis [4]. Furthermore, Ozturk et al. found that rats in the VC model group had significantly higher testis tissue hypoxia indices [5]. As a result of an anoxic environment, the body adjusts and changes. During tissue hypoxia, hypoxic inducible factor (HIF) is produced, and its level is proportional to the degree of hypoxia. Hypoxia-induced *HIF-1* can rapidly enter the nucleus and trigger transcription of *VEGF*, enabling cells to adapt to hypoxia. A study by Wang et al. found that VC on one side of testis could lead to hypoxia on the other side of testis, eventually causing spermatogenic cells to die [6]. The results of this study suggest that hypoxia promotes cell apoptosis, which can also be used to predict apoptosis of testicular germ cells, and is associated with increased cell apoptosis rate [7].

RNA modifications play an essential role in regulating gene expression posttranscriptionally. Eukaryotes have the most abundant internal modification, m^6^A, which constitutes 0.1%–0.3% of total adenosine residues [8, 9]. There is high conservation between humans and mice for m^6^A methylation modification. It is located in the non-coding 3′ term, near the stop codon and long internal exons, and is related to RNA stability, splicing, intracellular distribution, and translation [10, 11]. The cellular m^6^A state is controlled by a group of genes called “writers” (*WTAP*, *METTL3* and *METTLL4*), “erasers” (*FTO* and *ALKBH5*), and “readers” (*YTHDF1/2/3*, *IGF2BP2/3*, *YTHDC1* and *YTHDC2*) [12–18]. A multisubunit methyltransferase complex can increase m^6^A levels, while an m^6^A demethylase complex can reverse this process [8, 11, 19, 20]. N6-methyladenosine RNA modifications on the sixth nitrogen atom of adenine, one of the most important nitrogen atoms in RNA, have become one of the hottest topics in various human diseases, including hypertension [21], cardiac hypertrophy [22], viral infection [23], diabetes [24], and cancers [25, 26]. In another study, m^6^A modification was found to promote *PLOD2* protein translation after *YTHDF1* recognition, and then promote renal cancer development [27]. Yet, the expression patterns of RNA m^6^A methylation modification and their underlying mechanisms remain largely unknown in VC.

In the present study, we observed an increase in *PLOD2* m^6^A methylation and expression levels in the testes of VC rats compared with the control group. Preliminary experimental results showed that methylating the 3′UTR of *PLOD2* can regulate mRNA stability by recruiting m^6^A reader proteins. Furthermore, *PLOD2* demethylation promotes GC-2 cells apoptosis under oxidative stress.

## Methods

### Animals

20 adult male Wistar rats (180–200 g) were purchased from the Guangdong Provincial Center for Disease Control and Prevention. A 12 h/12 h light/dark cycle was maintained for the animals and they were fed standard food pellets and water as needed. The Institutional Animal Care and Use Committee approved all animal experiments in strict accordance with its guidelines.

### Establishment of a rat varicocele model

In an experimental study, twenty male Wistar rats were split into two distinct groups: the sham control group and the varicocele group. Anesthesia was induced with a solution containing 10 g of tribromoethanol and 10 mL of tert-pentyl alcohol, both from Sinopharm Chemical Reagent Co. (Shanghai, China), diluted to a 2% concentration in distilled water. A 2-cm surgical cut was initiated in the lower abdomen of each rat. The intersection of the left renal vein and the inferior vena cava was carefully isolated. A channel was created via blunt dissection between the inner side of the left renal vein and the exterior of the inferior vena cava. Subsequently, a 4-0 silk suture was employed to constrict the left renal vein to half its initial diameter. For the sham control group, an identical surgical procedure was followed, barring the vein constriction. The success of the modeling was gauged by two criteria: (i) a spermatic vein diameter greater than 1 mm, and (ii) the absence of any size disparity between the left and right kidneys. Eight weeks postoperation, the rats were euthanized for further analysis.

### RNA m^6^A and mRNA sequencing

As described previously, MeRIP-seq and RNA-seq were performed by Novogene (Beijing, China). Trizol (Thermo Fisher Scientific) was used to isolate total RNA from 5 pairs of tumors and adjacent tissues. Then total RNA was broken into almost 100 nt fragmentation and incubated with anti-m6A antibody (Synaptic Systems, 202003, Goettingen, Germany) for 2 h at 4 °C. The beads (Thermo Fisher Scientific) were then prepared and incubated with total RNA for 2 h at 4 °C. The final step is to wash the mixture and purify the m6A-bound RNA with TE buffer. Following purification, the samples can be used to construct the library using the NEBNext UltraTM RNA Library Prep Kit (New England Biolabs, MA, USA) on the Illumina HiSeq sequencer (Illumina, CA, USA). In the NCBI database, raw RNA-seq and m6A-seq data have been uploaded.

### Sequencing data analysis

To obtain the sequence data for IP and control samples, we should preprocess the read segment data (for example, filtering out the poor-quality segments), and a reference genome is used to process and analyze all the read segment sequence mapping of the two samples. As a result, read segments captured by methylation sites in the IP samples formed a region or peak near the methylation sites in the reference genome. It is therefore known as the peak-calling algorithm to identify points of methylation enrichment. We identified m6A methylated peaks among transcripts using Model-based Analysis of ChIP-Seq (MACS), and we investigated metagene m6A distribution using MetaPlotR. The DMGs were identified by diffReps. An analysis of Kyoto Encyclopedia of Genes and Genomes (KEGG) pathway enrichment and Gene Ontology (GO) was conducted on DMGs and DEGs from MeRIP-seq and RNA-seq.

### RNA-binding protein immunoprecipitation (RIP)

In accordance with the manufacturer’s instructions, RIP assays were performed with the Magna RIPTM RNA-Binding Protein Immunoprecipitation Kit (Millipore). A protease inhibitor cocktail and RNase inhibitor were included in the complete radioimmunoprecipitation assay buffer for lysing the cells. Protein A/G magnetic beads were prebound to antibodies (5 g) for 2 h before being incubated overnight with 100 L of cell lysate at 4 °C. By incubating the beads in 400 μL of elution buffer for 2 h, eluting with ethanol, and dissolving in RNase-free water, the RNA was eluted from the beads. Real-time PCR (qPCR) was used to determine fragment enrichment.

### RT-qPCR, RIP-qPCR and MeRIP-qPCR

Following the instructions of Vazyme Biotech, Nanjing, China, total RNA was extracted from tissues and cell lines using the RNA-easy Isolation Reagent. Anti-m6A antibody-coupled beads were used to incubate fragmented RNA. IGF2BP2 was used to immunoprecipitate the m6A-containing RNA and then it was eluted from the beads. The RT-qPCR was performed with gene-specific primers on both input control and m^6^A-IP samples. A HiScript III RT SuperMix for qPCR was used to synthesize the cDNA (Vazyme Biotech, Nanjing, China). With universal SYBR Green qPCR Master Mix (Vazyme Biotech, Nanjing, China) and spectrophotometry (ABI Prism 7500TM instrument, Applied Biosystems), qRT-PCR was performed.

### Protein isolation and western blot

KeyGEN Bio TECH protein extraction kit (KGP1100) was used to extract protein from cells and separate it on 10% SDS–PAGE and transfer it to nitrocellulose membrane. According to previous instructions, blots were then immunostained with primary antibodies and secondary antibodies. The antibodies were as follows: *PLOD2* (1:1000; Abcam, United States), *p-AKT* (1:1000; Abcam, United States), *AKT* (1:1000; Abcam, United States), *Bax* (1:1,000; Invitrogen, United States), *Bcl-2* (1:1000; Invitrogen, United States), *IGF2BP2* (1:1000; Invitrogen, United States), and *GAPDH* (1:10000; Proteintech, United States).

### Expression plasmids, short interfering RNAs, and lentivirus transfection

Addgene provided the CRISPR dCas13b plasmids and Cas13b-gRNA plasmids. A number of designed gRNAs and the dCas13b-ALKBH5 vector have been constructed by Synbio Technologies. An overexpression plasmid was generated using the CDS of *ALKBH5* cloned into pcDNA3.1. The vector control used for analysis was pcDNA3.1. *IGF2BP2* was knocked down with Sigma-synthesized duplex RNAi oligos.

### Cell culture and plasmid transfection

The GC-2 cell line for this study was purchased from the American Type Culture Collection (ATCC, Manassas, VA, United States). DMEM with 10% fetal bovine serum was routinely used for cell culture, supplemented with 5% CO_2_ in a 37 °C environment (Invitrogen, Carlsbad, CA, United States). Lipo3000 (Invitrogen) was used for transfection of all siRNAs and plasmids as per the manufacturer’s instructions, and 1 μg of plasmids was used in each experiment. There was a 50 nM working concentration of siRNA.

### Luciferase reporter assay

To determine the effect of *PLOD2* expression, *PLOD2*’s wild type or mutant 3′UTR was inserted at the end of the firefly luciferase (F-luc) coding sequence. In both wild type and *ALKBH5* overexpression cells, the pmirGLO-*PLOD2*-3′UTR-WT and pmirGLO-*PLOD2*-3′UTR-MUT were transfected for 24 h. We analyzed firefly luciferase (F-luc) and renilla luciferase (R-luc) by using the Dual-Glo Luciferase Assay system (Promega) according to instructions. The activity of Renilla Luciferase (R-luc) was used to normalize the activity of Firefly Luciferase (F-luc) to evaluate reporter transcription. The experiments were repeated three times and the results were similar each time.

### mRNA stability

Cells transfected with different plasmids were stabilized with actinomycin D (Act-D, catalog #A9415, Sigma, U.S.A.) at 50 mg/mL during incubation. To conduct real-time PCR, RNA was isolated from the cells at the indicated times. PLOD2 mRNA half-life was calculated by using ln2/slope and normalized using *GAPDH*.

### Cell proliferation and apoptosis assays

Detection of cell proliferation was carried out using the Cell Counting Kit 8 (CCK8) assay (Transgen, China). In 96-well plates, 3 × 10^3^ cells were seeded per well. A total of 24 h, 48 h, 72 h, and 96 h of cell culture was followed by 3 h of incubation with CCK8 at 37 °C. After that, 450 nm absorbance was measured using a microplate reader. Approximately 70%–80% confluence was achieved with vector-transfected cells plated in a 12-well plate (2 × 10^5^ /well). Flow cytometry assays were performed 48 h after cell harvesting.

### Statistical analyses

At least three independent experiments were used to gather data. Data are reported as mean ± standard deviation. A two-tailed unpaired Student’s *t*-test was used between two groups, along with one-way or two-way ANOVA and Bonferroni testing for multiple comparisons. A two-sided test was used for all statistical analyses, using SPSS 16.0 for Windows. To be considered statistically significant, the *p*-value must be 0.05 or less, e.g., *p < 0.05, **p < 0.01. “NS” stands for not significant.

## Results

### Distribution of m^6^A modification in the testes of VC rat and control group

**Fig. 1:**
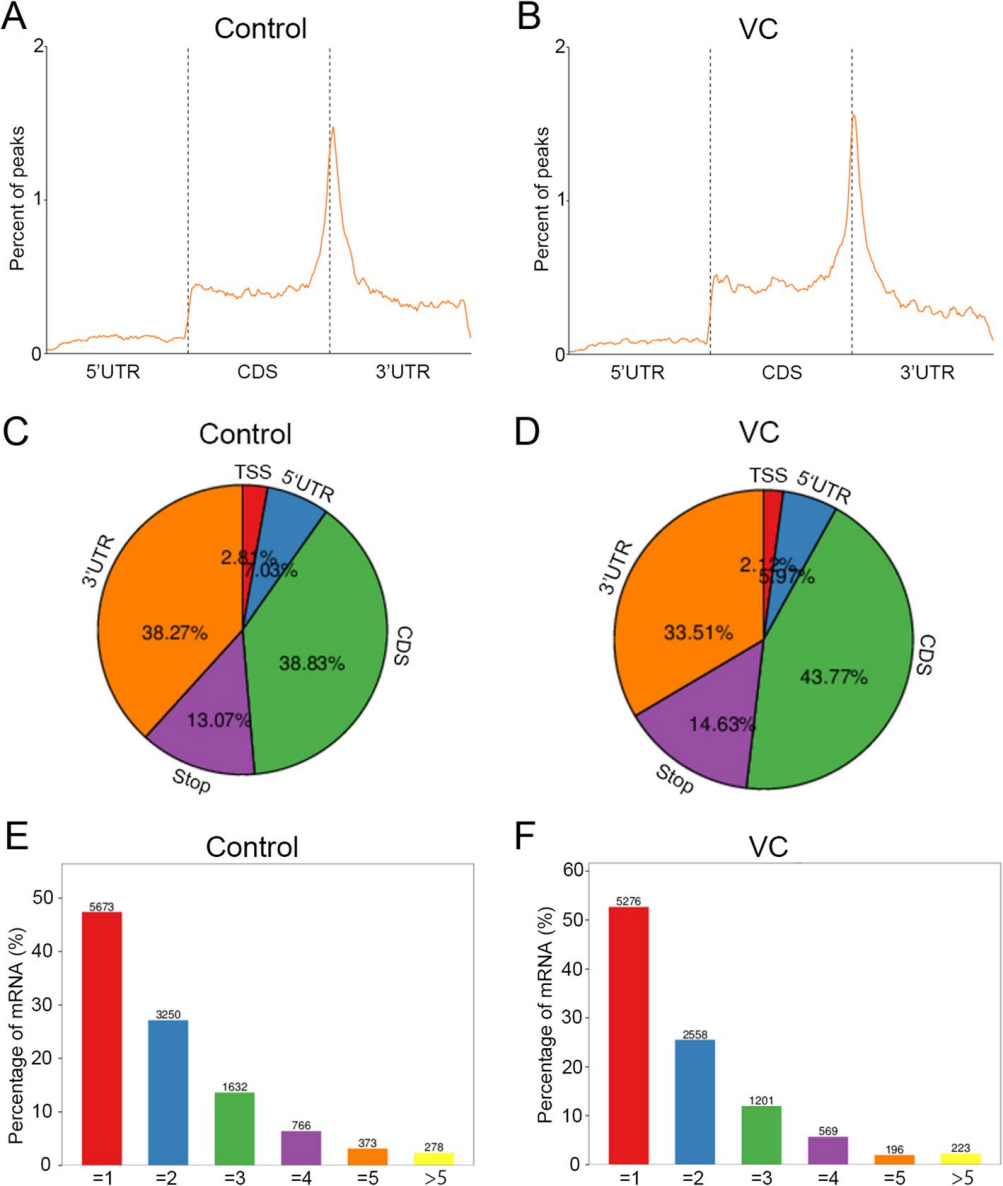
Distribution of m^6^A modification in the testes of VC rat and control group. **A, B** A metagene profile of transcript peaks in the control (**A**) and VC (**B**) groups. **C**, **D** A measure of the proportion of m^6^A peaks across the entire transcriptome of control and VC groups (**C** and **D**). **E**, **F** The mRNAs in control (**E**) and VC (**F**) groups harboring different numbers of m^6^A peaks

To understand the mechanisms of m^6^A methylation modification in the testes of VC rats in greater detail, whole testicular samples were used for mRNA m^6^A methylation sequencing (MeRIP-seq). First, we analyzed the metagene profiles of transcript peaks in the testes of control and VC rats. We found that the majority of m^6^A peaks were situated at the end of the 5′UTRs and start of the 3′UTRs in two groups (Fig. 1A and B). Further analysis revealed that peaks located at coding sequence (CDS) were the most frequent, and peaks located at translation start site (TSS) were the least frequent in both groups (Fig. 1C and D). Afterwards, the rat transcriptome was analyzed for their m^6^A methylation modifications. In VC and control groups, most of the methylated sequences within mRNA contained less than five m^6^A peaks, while few had more than five (Fig. 1E and F). Similar results were found in previous studies for the distribution of m^6^A modifications.

### Functional analysis of differentially m^6^A methylated mRNAs between two groups

**Fig. 2:**
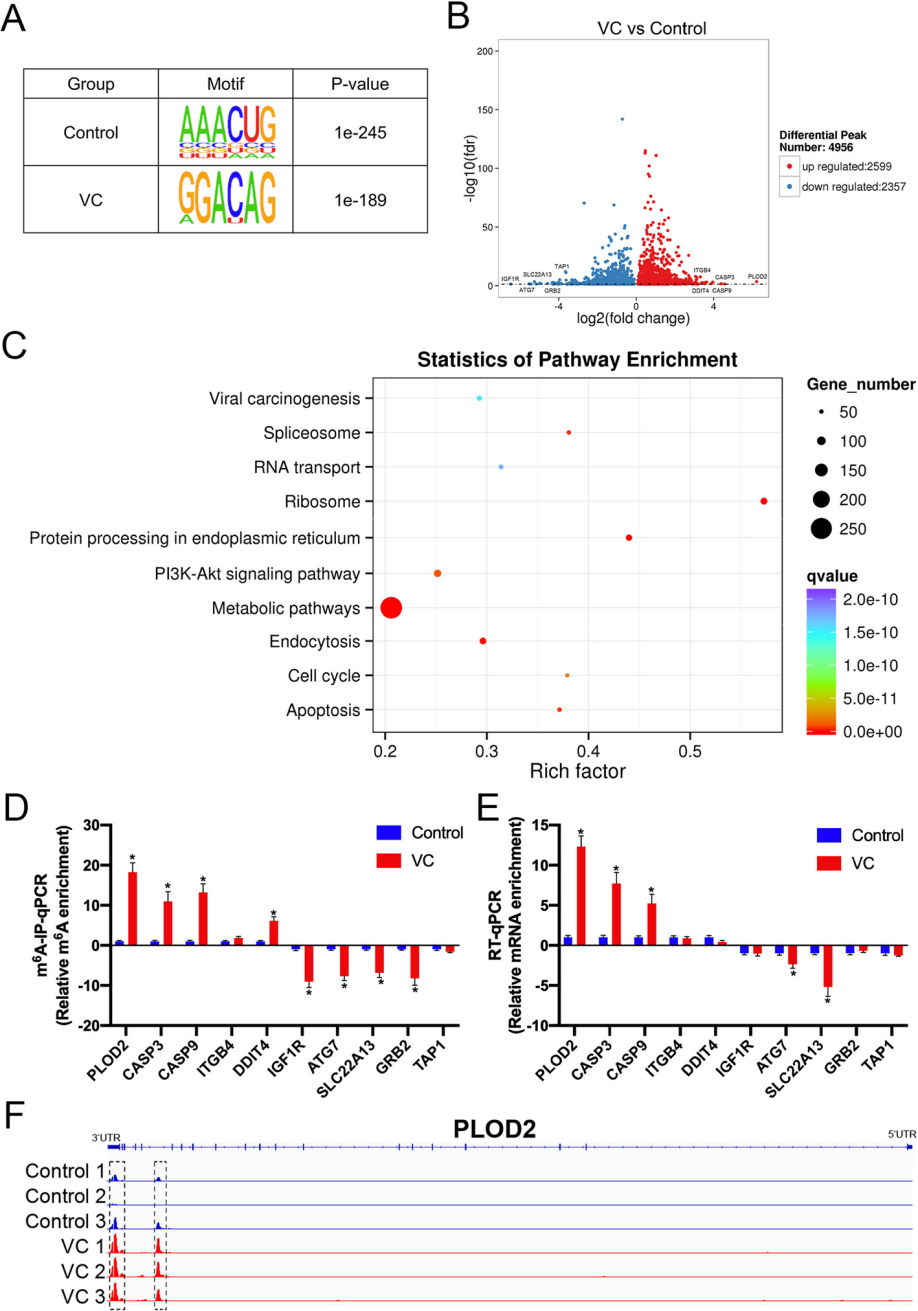
Functional analysis of differentially m^6^A methylated mRNAs between two groups. **A** An analysis of sequence motif among peak regions that contain m^6^A site in both control and VC groups. **B** Volcano plots showing differentially m^6^A-modified peaks in mRNAs based on |log2FC| > 1 and *p*-value < 0.05. In volcano plots, red blots represent hypermethylation and blue blots represent hypomethylation. **C** An analysis of the KEGG pathway of mRNAs that contain differentially methylated m^6^A sites. **D** MeRIP-qPCR analysis validates m^6^A enrichments for five hypermethylated genes and five hypomethylated genes. **E** RT-qPCR analysis validates mRNA expression levels for five upregulated genes and five downregulated genes. **F** According to the Integrative Genome Viewer (IGV) software, *PLOD2* mRNA showed different m^6^A methylation patterns in the VC and control groups

To determine whether the m^6^A peaks we identified from MeRIP-seq represented the RRACH motif, we performed the HOMER motif software analysis on the m^6^A peaks. The motif sequences in the control and VC groups were AAACU and GGACA, respectively (Fig. 2A). Based on |log2FC| > 1 and *p*-value < 0.05, the differentially methylated m^6^A peaks were identified between the control and VC groups. As compared with the control group, 2599 hypomethylated peaks and 2357 hypermethylated peaks were found in the VC group (Fig. 2B). As a result of KEGG pathway analysis, differentially m^6^A methylated peaks within mRNA were predominantly associated with metabolic pathways, PI3K-Akt signaling pathways, and cell apoptosis (Fig. 2C). MeRIP-qPCR assays were performed to confirm our MeRIP-seq results for five hypermethylated genes (*PLOD2*, *CASP3*, *CASP9*, *ITGB4*, and *DDIT4*) and five hypomethylated genes (*IGF1R*, *ATG7*, *SLC22A13*, *GRB2*, and *TAP1*), which were potentially involved in cell apoptosis and the *PI3K-Akt* signaling pathway. These genes exhibited almost the same m^6^A-level changes with sequencing, supporting our MeRIP-seq results (Fig. 2D). We also performed RT-qPCR analysis on VC and control groups to determine the transcript levels of these genes (Fig. 2E). Results showed that *PLOD2*, *CASP3*, and *CASP9* were upregulated, and *ATG3* and *SLC22A13* were downregulated in the VC group compared with the control group. As a result, *PLOD2* was the gene with the highest level of methylation and expression in VC group. Based on these results, we investigated the general locations of differentially methylated m^6^A sites within *PLOD2* in VC group and control group. Our data indicated that the m^6^A peak was enriched around *PLOD2*’s 3′UTR in two groups, and it was higher in the VC group than in the control group (Fig. 2F). In summary, we found *PLOD2* was hypermethylated and upregulated in the testes of VC rats.

### *PLOD2* expression and m^6^A methylation level under oxidative stress in GC-2 cell

**Fig. 3:**
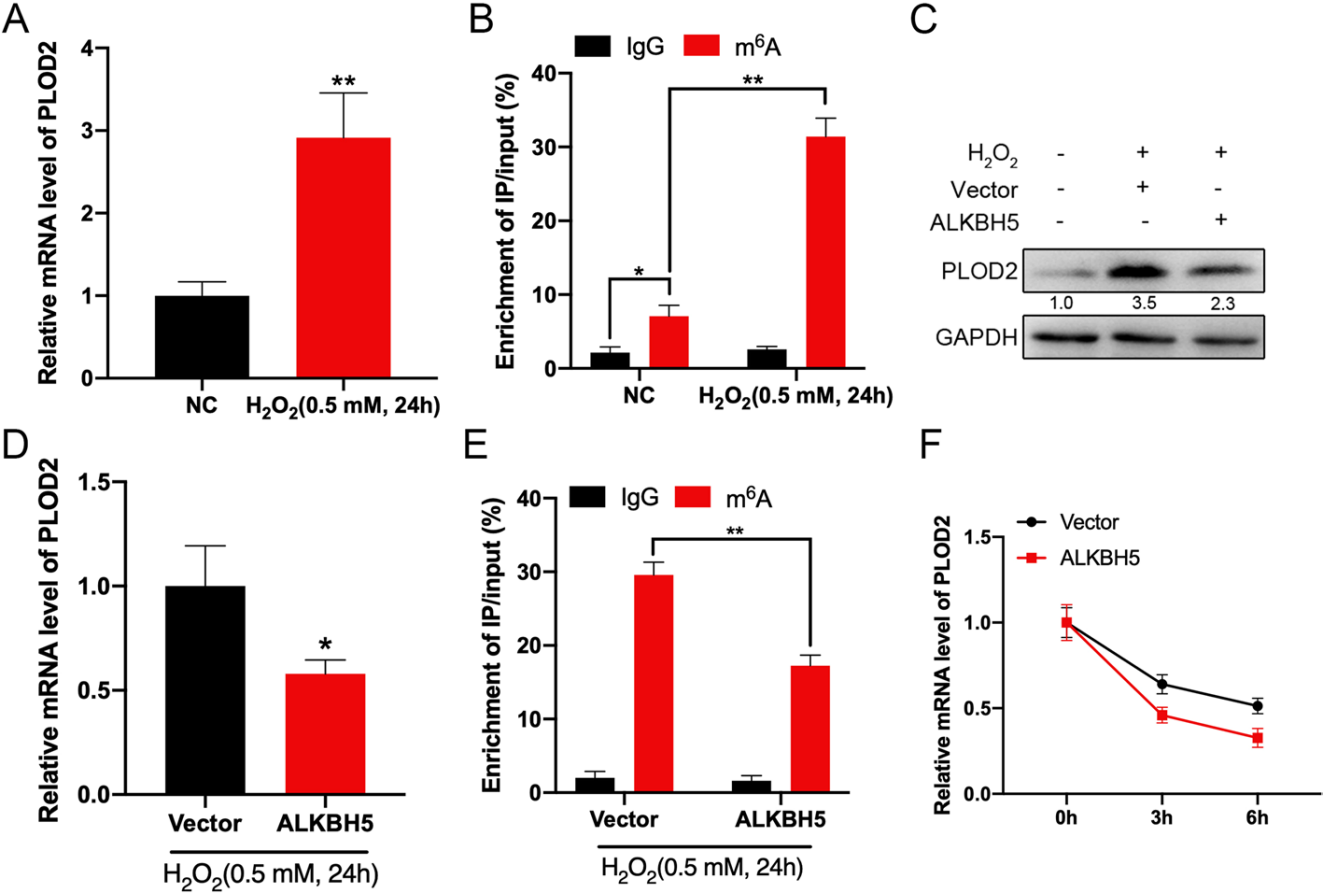
*PLOD2* expression and m^6^A methylation level under oxidative stress in GC-2 cell. **A** Under oxidative stress, the mRNA expression levels of *PLOD2* in GC-2 cell were detected using RT-qPCR. **B** Under oxidative stress, the m^6^A methylation levels of *PLOD2* in GC-2 cell were detected using MeRIP-qPCR. **C**, **D** Western blot (**C**) and RT-qPCR (**D**) were used to measure *PLOD2* protein and mRNA expression levels in GC-2 cells transfected with vector control or *ALKBH5* construct for 24 h under oxidative stress. **E** MeRIP-qPCR analysis of *PLOD2* m^6^A levels in control and overexpression of *ALKBH5* in GC-2 cells under oxidative stress. **F** The mRNA levels of *PLOD2* were checked for the indicated times in control and *ALKBH5*-overexpressed GC-2 cells after treatment with Act-D

For further investigation of how m^6^A methylation modification regulates *PLOD2* expression in varicocele, the spermatocyte cell line GC-2 was used. To mimic varicocele’s oxidative stress, H_2_O_2_ (0.5 mM, 24 h) was added to the GC-2 cell culture medium. According to previous studies, H_2_O_2_ reduced the viability of GC-2 cells in culture in a dose-dependent manner [28]. In comparison with control cells, GC-2 cells expressed higher levels of *PLOD2* mRNA after H_2_O_2_ treatment (Fig. 3A). In addition, m^6^A enrichment of *PLOD2* was also significantly greater in GC-2 cells treated with H_2_O_2_ than in control cells, as determined by MeRIP-qPCR (Fig. 3B).

Next, we investigated the mechanisms via which m^6^A modification affects *PLOD2* expression in GC-2 cell. Results of western blots and RT-qPCR analysis showed that after H_2_O_2_ treatment, *ALKBH5*-overexpressing GC-2 cells expressed less *PLOD2* protein and mRNA (Fig. 3C and D). In addition, when GC-2 cells were treated with H_2_O_2_, overexpression of *ALKBH5* significantly inhibited m^6^A antibody-enriched *PLOD2* mRNA (Fig. 3E and Additional file 1: Fig. S1A). Additionally, Act-D was used to block transcription in normal control and *ALKBH5*-overexpressing GC-2 cells. It has been shown that *PLOD2* mRNA half-lives are shortened by overexpression of *ALKBH5* (Fig. 3F). As a result, m^6^A modification might delay *PLOD2* mRNA degradation in GC-2 cells.

### *IGF2BP2* affected the mRNA stability of *PLOD2* by participating in the *PLOD2* m^6^A modification

**Fig. 4:**
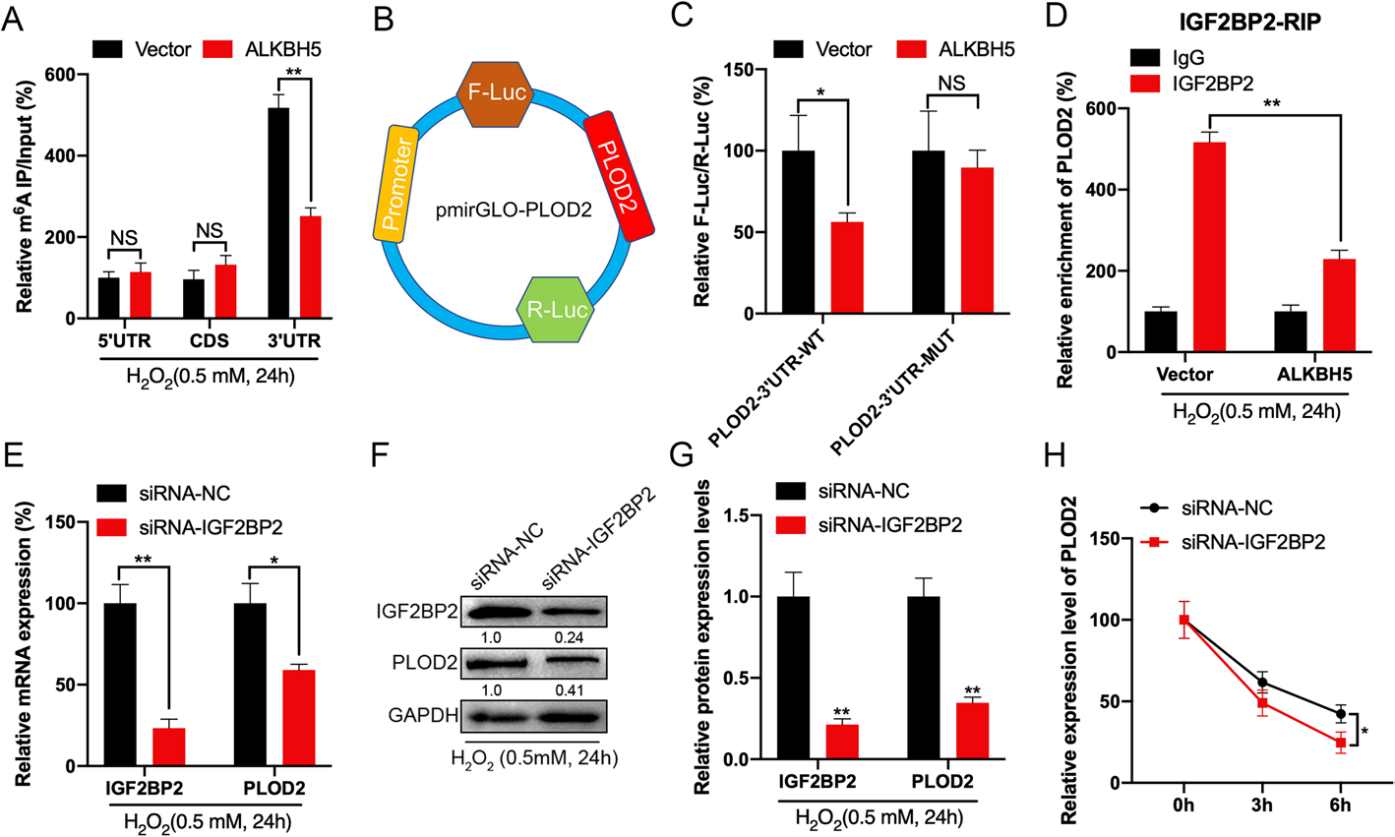
*IGF2BP2* affected the mRNA stability of *PLOD2* by participating in the *PLOD2* m^6^A modification. **A** By using fragmented RNA, MeRIP-qPCR was used to analyze the enrichment of m^6^A in 5′UTR, CDS, or 3′UTR of *PLOD2* in control or *ALKBH5* overexpression GC-2 cells under oxidative stress. **B**, **C** In control and *ALKBH5*-overexpressing GC-2 cells, the relative luciferase activity of F-Luc/R-Luc of pmirGLO-*PLOD2*-3'UTR-WT and the relative activity of F-Luc/R-Luc of pmirGLO-3′UTR-MUT were determined. **D** *PLOD2* mRNA was analyzed by RIP-qPCR using *IGF2BP2* antibody in control or *ALKBH5*-overexpressing GC-2 cells. **E**–**G** In GC-2 cells transfected with control or siRNA-*IGF2BP2* siRNAs, *IGF2BP2* and *PLOD2* mRNA (**E**) and protein (**F** and **G**) expression levels were detected by RT-qPCR and western blotting. **H** The mRNA of *PLOD2* was determined by RT-qPCR after transfecting GC-2 cells with control siRNA or siRNA-*IGF2BP2* for 24 h and then further treating them with Act-D

In *PLOD2* mRNA, several differentially methylated m^6^A peaks (DMMPs) were detected by MeRIP-seq (Fig. 1F). With an anti-m^6^A antibody, fragmented RNA from GC-2 cells was immunoprecipitated to characterize m^6^A methylation. According to MeRIP-qPCR results, the 3′UTR exhibited the highest level of m^6^A methylation, followed by the CDS and the 5'UTR region (Fig. 4A). Thus, in GC-2 cells overexpressing *ALKBH5*, m^6^A enrichment of *PLOD2* 3'UTR was decreased, which indicated m^6^A modifications are more dynamic in the 3'UTR than in the CDS (Fig. 4B). Based on luciferase assays in GC-2 cells using reporters containing *PLOD2*-3′UTR-WT or -MUT, we investigated the potential role of m^6^A methylation in the 3′UTR region of *PLOD2* (Fig. 4B). According to the dual-luciferase assay, *PLOD2*–3’UTR-WT translated more efficiently in GC-2 cells overexpressing *ALKBH5* compared with controls (Fig. 4C).

Further investigations were conducted into mechanisms regulating mRNA stability by m^6^A methylation modification. Researchers found that m^6^A modification regulates mRNA stability by binding to *YTHDF2*, *YTHDF3*, and *IGF2BP1 ~ 3*. Therefore, we explored which m^6^A reader protein enhances *PLOOD2* mRNA stability in a manner dependent on m^6^A modification. To determine whether *IGF2BP2* binds significantly to *PLOD2* mRNA in GC-2 cells, RIP-qPCR assays were performed. Results showed *IGF2BP2* bound significantly to *PLOD2* mRNA (Fig. 4D and Additional file 1: Fig. S1B). Thus, we knocked down *IGF2BP2* in GC-2 cells and found that *IGF2BP2* downregulation inhibited the mRNA and protein levels of *PLOD2* (Fig. 4E–G). It has also been shown that downregulation of *IGF2BP2* can decrease the stability of *PLOD2*’s mRNA (Fig. 4H). Based on these results, *IGF2BP2* may be involved in the m^6^A methylation modification and in regulating the stability of *PLOD2* mRNA.

### Targeting m^6^A demethylation of *PLOD2* by CRISPR/dCas13b-ALKBH5 to regulate GC-2 cell proliferation and apoptosis under oxidative stress

**Fig. 5:**
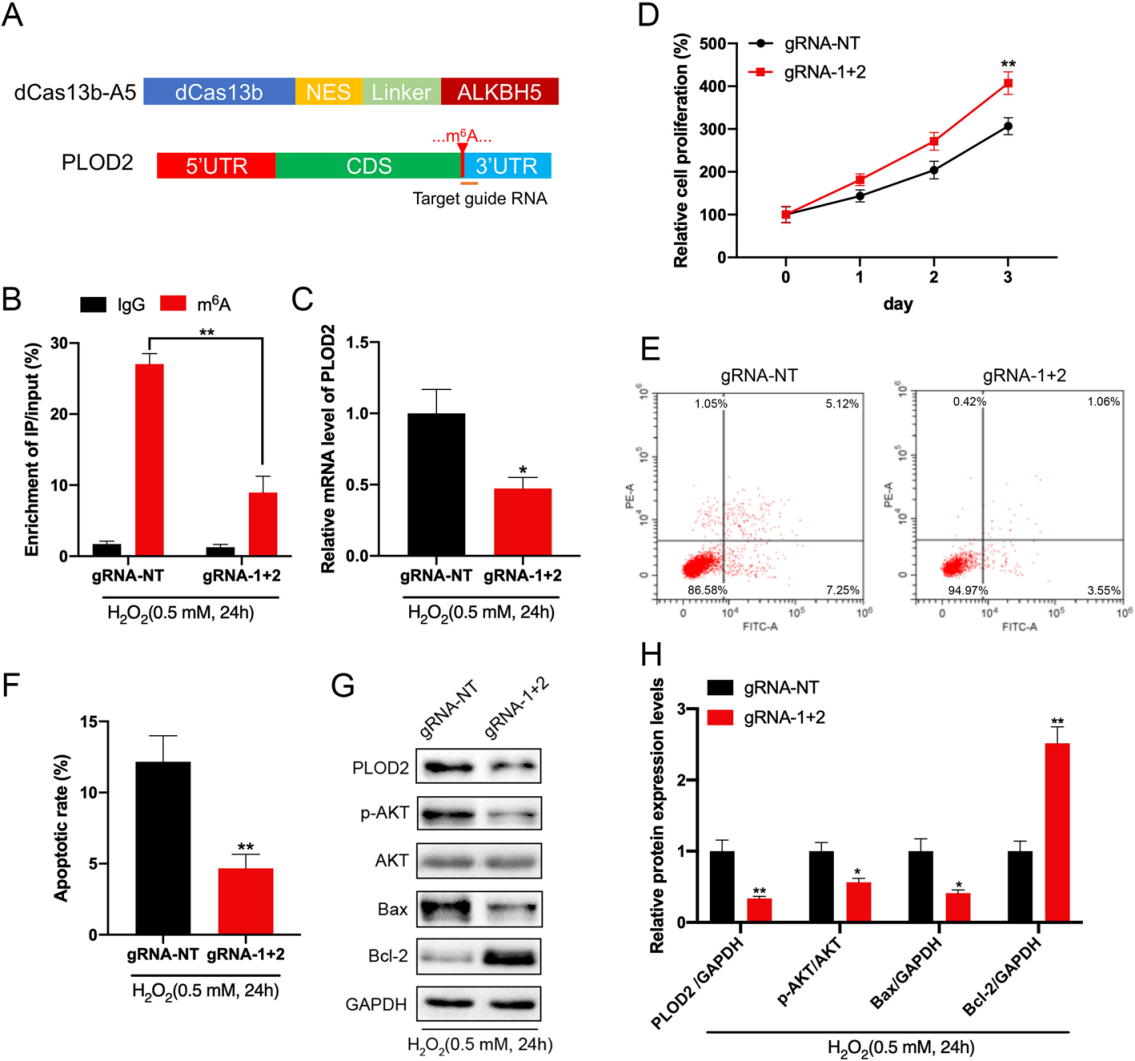
Targeting m^6^A demethylation of *PLOD2* by CRISPR/dCas13b-ALKBH5 to regulate GC-2 cell proliferation and apoptosis under oxidative stress. **A** Figure depicts the position of the m^6^A site within *PLOD2* mRNA and the target guide RNA’s target regions. **B**, **C** Under oxidative stress, m^6^A (**B**) and mRNA (**C**) levels of *PLOD2* were measured in GC-2 cells transfected with dCas13b-ALKBH5 with a gRNA control or a gRNA for *PLOD2*. **D** GC-2 cells transfected for 24 h with dCas13b-ALKBH5 in combination with gRNA control or gRNA for *PLOD2* are evaluated for cell proliferation using the CCK8 assay. **E**, **F** Transfection of GC-2 cells with dCas13b-ALKBH5 together with control gRNA or gRNA for *PLOD2* to investigate cell apoptosis. **G**, **H** Under oxidative stress, GC-2 cells transfected with dCas13b-ALKBH5 combined with gRNA control or gRNA for *PLOD2* for 24 h were examined for protein levels of *PLOD2*, *p-AKT*, *Bax*, and *Bcl-2*

In the next step, we demethylated the m^6^A of *PLOD2* by fusing the catalytically dead type VI-B Cas13 enzyme with the m^6^A demethylase *ALKBH5* (dCas13b-A5) enzyme [29]. To target the PLOD2 mRNA, specific guide RNAs were designed around the m^6^A site (Fig. 5A). The dCas13b-A5 induced demethylation of *PLOD2* in GC-2 cells was first confirmed by MeRIP-qPCR (Fig. 5B). As a result of dCas13b-A5 targeting *PLOD2*, we observed a significant decrease in *PLOD2* mRNA and protein levels in GC-2 cells (Fig. 5C). There might be a reason for this, since dCas13b-A5 with a gRNA for *PLOD2* significantly decreases *PLOD2* mRNA binding to *IGF2BP2* protein. To investigate whether dCas13b-A5 targeting *PLOD2* can modulate GC-2 cell homeostasis, we monitored GC-2 cell proliferation and apoptosis after transfection with control or gRNA for *PLOD2* combined with dCas13b-A5. Compared with non-targeted control gRNA combined with dCas13b-A5, gRNA targeting *PLOD2* significantly promoted cell proliferation and decreased apoptosis in GC-2 cells (Fig. 5D–F). In addition, western blot analysis showed that *PLOD2*-dymethylated GC-2 cells had decreased Bax protein level and increased Bcl-2 level. In GC-2 cells, p-Akt expression was also decreased after the *PLOD2* mRNA demethylation (Fig. 5G, H). These results suggest that a decreased m^6^A methylation level of *PLOD2* can promote cell proliferation, reduce cell apoptosis, and further inactivate the PI3K/AKT/mTOR pathway.

## Discussion

Varicocele is the abnormal expansion and tortuosity of the tendriform venous plexus in the spermatic vein, with the left side being the most common [30]. It was first described by Saypol et al. nearly a century ago that partial ligation of the left renal vein simulating varicocele could be performed. Hence, the left renal vein was partially ligated in this study to create the VC model. VC has been proved by a number of studies to cause serious damage to testicular spermatogenic function, which can then cause male infertility. The main pathophysiological cause of VC is persistent hypoxia at the site of testicular spermatogenesis, as oxygen is essential for spermatogenic tubules to produce normal spermatogenesis [31]. As a result of VC, the one-way blood flow system between the spermatic vein and the testicular vein is damaged, resulting in venous blood stasis. As a result, it causes the hydrostatic pressure inside the testicle to increase and overtake the microcirculation pressure inside the testicular artery, causing a relatively anoxic situation [32]. Hypoxia in testicular tissue can inhibit HIF-1α protein degradation, leading to increased expression levels and downstream gene activation [33, 34]. It has also been demonstrated that hypoxia controls sarcoma metastasis by enhancing the expression of the intracellular enzyme *PLOD2* [35].

In eukaryotes, the dynamic and reversible m^6^A RNA modification is mediated by methyltransferases and demethylases. In addition to heat shock [36], ultraviolet light [37], hypoxic stress [38], and oxidative stress [39], m^6^A modification participates in many cellular activities and reactions. Although numerous studies have confirmed the hypoxic stabilization of specific mRNAs is dependent on m^6^A modification, less is known about their role in VC. By activating hydroxylation of collagen fiber molecules, *PLOD2* plays an important role in fibrotic processes and tissue remodeling [40]. Overexpression of *PLOD2* can lead to collagen crosslinking, an increase in extracellular matrix hardness, and cancer cell proliferation and metastasis [41]. However, whether m^6^A methylation modification could influence *PLOD2* expression to alter epigenetic remodeling, or contribute to the features of VC, is unclear and worthy of investigation.

Based on the results from this study, *PLOD2* is likely to be a modulatory biomarker in varicocele. Based on MeRIP-seq technology, we identified many differentially methylated genes among VC and control rats and found that *PLOD2* was frequently upregulated in VC groups. Furthermore, our data indicate that activation of *PLOD2* might be due to an increase in m^6^A methylation modification in the 3′UTR regions of mRNA in the VC group. When *ALKBH5* is overexpressed in GC-2 cells, it can decrease m^6^A methylation level, thus decreasing *PLOD2* mRNA stability and expression levels.

In addition to RNA processing, nuclear export and translation modulation, the m^6^A modification can regulate many stages of RNA’s life cycle [42, 43]. According to our findings, the luciferase reporter system showed that m^6^A in the 3′UTR of mRNA positively regulated *PLOD2* m^6^A methylation modification. Furthermore, m^6^A-regulated mRNA stability of *PLOD2* was dependent on *IGF2BP2* in GC-2 cells. It has been demonstrated that *IGF2BPs* can bind the GG(m6A)C sequence of mRNA to promote its stability and storage [44]. Overall, our data provide a new insight into the function of *IGF2BP2* regulating *PLOD2* mRNA stability under hypoxic stress. A newly developed method called CRIPSR/dCas13b-ALKBH5 system regulates m^6^A modification level of target genes by targeting demethylation of specific mRNA in the transcriptome [29]. With CRIPSR/dCas13b-ALKBH5, we specifically demethylated the m^6^A modification of *PLOD2* mRNA. m^6^A methylation level was decreased about two-fold and *PLOD2* expression was also significantly decreased by this system. In GC-2 cells, dCas13b-A5 promoted cell proliferation while inhibiting apoptosis and inactivating *PI3K/AKT/mTOR* signaling.

## Conclusion

In summary, we found that m^6^A methylation modification played va ital role in oxidative stress-induced apoptosis in varicocele, and may be used as a novel target for oxidative stress-related male infertility. Furthermore, the regulatory network involving the new complex *ALKBH5/PLOD2* may provide insight into the pathogenesis and development of male infertility.

### Supplementary Information

**Additional file 1: Figure S1.** Factors involved in m^6^A-regulated expression of *PLOD2*. **A** MeRIP-qPCR analysis of *PLOD2* m^6^A levels in control and overexpression of *ALKBH5* or *FTO* cells. **B** RIP-qPCR analysis of *PLOD2* enrichment levels using *IGF2BP1*, *IGF2BP2*, and *IGF2BP3* cells

## Data Availability

The datasets used and/or analyzed during the current study are available from the corresponding author on reasonable request.

## Abbreviations

m^6^A: N6-methyladenosine
MeRIP-seq: M^6^A methylated RNA immunoprecipitation-sequencing
RNA-seq: RNA sequencing
3′-UTR: 3′ Untranslated region
VC: Varicocele
HIF: Hypoxic inducible factor
5′-UTR: 5′ Untranslated region
CDS: Coding sequence
TSS: Translation start site
DMMPs: Differentially methylated m^6^A peaks
