# Supplementary figures and images for: N6-methyladenosine modification of PLOD2 causes spermatocyte damage in rats with varicocele
Source: Cell Mol Biol Lett. 2023 Sep 5;28:72. doi: 10.1186/s11658-023-00475-4 (PMC10481479; doi:10.1186/s11658-023-00475-4)

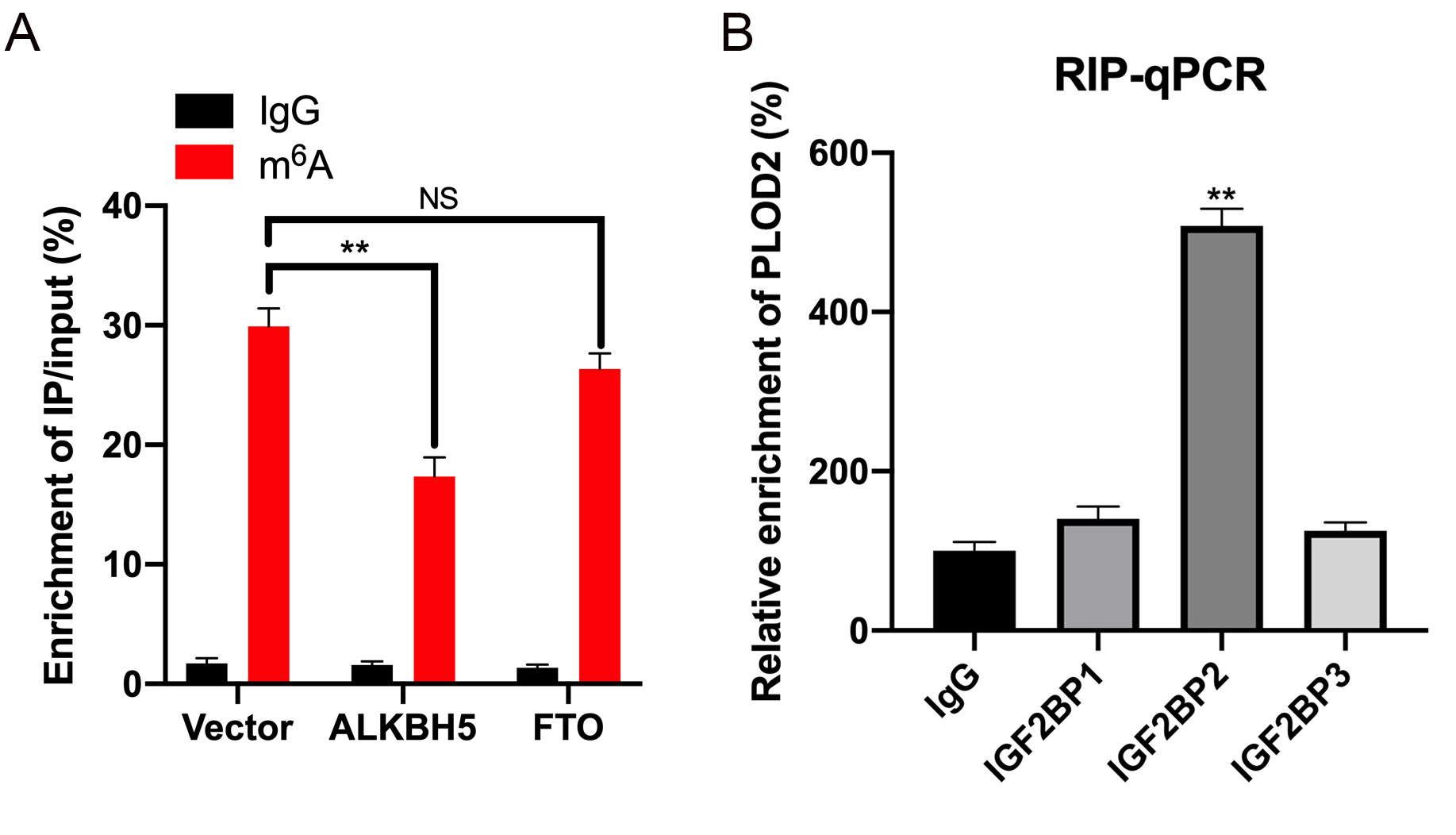

Supplement: Supplementary file 1 — Additional file 1: Figure S1. Factors involved in m6A-regulated expression of PLOD2. A MeRIP-qPCR analysis of PLOD2 m6A levels in control and overexpression of ALKBH5 or FTO cells. B RIP-qPCR analysis of PLOD2 enrichment levels using IGF2BP1, IGF2BP2, and IGF2BP3 cells [file 11658_2023_475_MOESM1_ESM.jpg]
